# Google Trends in Breast and Cervical Cancer Searches From 2008 to 2021: An Infodemiology Study

**DOI:** 10.7759/cureus.39035

**Published:** 2023-05-15

**Authors:** Akshaya S Bhagavathula, Surbhi Bansil, Yoshito Nishimura

**Affiliations:** 1 Center for Public Health and Technology, Health, Human Performance and Recreation, University of Arkansas, Fayetteville, USA; 2 Department of Medicine, University of Hawaii John A. Burns School of Medicine, Honolulu, USA

**Keywords:** public interest, google trends, awareness, infodemiology, cervical cancer, breast cancer

## Abstract

Introduction: Breast and cervical cancer are the leading causes of cancer death among women worldwide. Given the growing concern, cervical cancer awareness month (CCAM) in January and Breast cancer awareness month (BCAM) in October occur annually as global health observances to raise public awareness. This infodemiology study aimed to assess trends in public online searches for breast cancer and cervical cancer following the annual BCAM and CCAM from 2008 to 2021.

Methods: Google Trends (GT) was used to investigate online searches for "breast cancer" and "cervical cancer" from January 1, 2008, to December 31, 2021. (168 months). A joinpoint regression analysis was used to identify statistically significant weekly percentage changes (WPCs) and monthly percentage changes (MPCs) trends over time.

Results: Breast cancer searches increased in October (BCAM) every year, while cervical cancer searches increased in January (CCAM) in 2013, 2019, and 2020. Joinpoint regression analysis revealed a significant negative trend in "breast cancer" searches from 2008 to 2021 (MPC: -0.2%, 95% CI: -0.3 to -0.1) and an upward trend in "cervical cancer" searches from May 2017 to December 2021 (MPC: 0.5%, 95% CI: 0.2 to 0.7).

Conclusion: Online searches on "breast cancer" remain consistently high only during BCAM, and "cervical cancer" has increased by 0.5% MPC since May 2017. Our findings can inform online interventions like event-based opportunities (BCAM and CCAM) and Google Ads to raise public awareness of breast and cervical cancer.

## Introduction

Cancer is a leading cause of death worldwide. The most commonly diagnosed cancers and causes of cancer death in women worldwide are breast and cervical cancer [1]. There is a tremendous global burden of breast cancer, which is the most common and leading cause of cancer death in women worldwide [2]. An estimated 2.3 million new cases were diagnosed, and 684,000 deaths from breast cancer occurred in 2020 alone [3]. Disparities in prognosis have been demonstrated among regions across the world. Based on 2020 GLOBOCAN data, the highest incidence rates of breast cancer were observed in high-income regions such as Australia, New Zealand, Western Europe, North America, and Northern Europe, whereas the highest mortality rates were observed in Melanesia, Western Africa, Micronesia, and Polynesia [1].

Additionally, the CONCORD-3 study showed marked disparities in five-year survival rates from breast cancer, with high-income countries such as the United States being 90.2%, whereas low-income countries such as India being as low as 66.1% [4]. Much of these outcome disparities have been attributed to differences in accessibility to population-wide mammographic screening, preventive public education on breast health, diagnostic services (i.e., clinical evaluation, imaging, surgical sampling, and pathology), and multidisciplinary cancer treatment (i.e., systemic therapy, radiation therapy, and surgery) [2]. This can be attributed to a lack of public awareness of breast cancer; as many as 84% of women are aware of breast cancer, but significantly fewer women have in-depth knowledge of symptoms and risk factors, with a rate as low as 16% in low-income countries [5]. Because of variations in public awareness, variations in rates of preventive screening with mammography have also been observed in various regions worldwide. For instance, screening rates are as high as 94% in high-income countries such as Sweden to as low as 18% in low- and middle-income countries (LMICs) such as Mexico [6].

One means of increasing breast cancer prevention and public awareness has been the annual Breast Cancer Awareness Month (BCAM), which takes place in October. This campaign was initially pioneered in the United States in 1985 by the American Cancer Society and the American Academy of Family Physicians. It has been considered a successful campaign in increasing the early detection of breast cancer through mammography in the United States and has since been adopted by other countries across the world in response to the growing breast cancer burden [7,8].

Cervical cancer is also prominent among women, being the fourth most common cancer among women globally [1]. With more than 95% of cases due to human papillomavirus (HPV), cervical cancer is essentially considered preventable through vaccination against HPV and appropriate screening [9,10]. As with breast cancer, disparities in public awareness and access to preventive means have contributed to disparities in health outcomes from cervical cancer across the world. In 2020, greater than 90% of new cervical cancer and cancer deaths occurred in LMICs, likely due to the considerable disparity in HPV vaccination rates compared to high-income countries stemming from vaccine hesitancy [1,11-13]. The annual Cervical Cancer Awareness Month (CCAM) in January, established by the United States National Cervical Cancer Coalition (NCCC), a program of the American Sexual Health Association (ASHA), has been used as a means to increase public awareness [14].

While BCAM and CCAM were originally proposed in the United States, they are now recognized as worldwide health observances [15,16]. To date, it is unclear how effective BCAM and CCAM are worldwide in increasing public awareness of breast and cervical cancers. We aimed to assess trends in public online searches for breast and cervical cancer following the annual BCAM and CCAM using global Google Trends (GT) data from 2008 to 2021.

## Materials and methods

Google Trends

GT is a web-based online tracking system that provides information and trends about Google search volumes. GT is a freely available data source based on Google search data that has been widely used to investigate public interest and health-seeking behavior. GT has been utilized in public health research as it enables us to quantify the popularity or interest of a specific search query using Google search volumes as surrogates, as shown in a number of previous literature. The use of GT has been proposed as a way to narrow information gaps for low-income countries, and applying the GT analysis to low-income countries is still considered valid [17].

GT enables the exploration of online searches at different time intervals and retrieves the popularity of queries for any keywords entered in the Google search engine. Search queries in GT can be performed using the "search term" and "search topics" options. "Search term" provides the results of all keywords that fall within categories, and "search topic" renders the results of a group of terms that share the same concept in any language. Analyzing GT data provides us with the ability to determine the relative popularity of a specific search term in a particular category (example: "health"), place, and time range, suggesting how popular the search term is at a specific time and location. Relative popularity is noted as relative search volumes (RSV) on a scale of 0 (minimal or no interest) to 100 (high popularity) based on the term's search volume [18]. RSV is measured in two ways [19]. First, the popularity of a specific search term in a given week relative to other weeks in the designated period within a geographic area. The most popular week is defined as having an RSV of 100, and all other weeks’ RSVs are defined relative to the most popular week on a scale from 0 to 99. Second, the popularity of search terms over the designated period can be compared between geographic regions. The region with the highest search proportion is defined to have an RSV of 100, and other RSVs are given in relation to the highest.

Moreover, GT also provides top and rising queries. Top queries are the most popular queries within the used search parameters, and these queries tend to stay relatively consistent across time periods. On the contrary, rising queries tend to increase in terms of relative interest, and the interest is expressed in percentages. An increase in popularity above 5000% indicates a "Breakout".

Data collection

**Figure 1:**
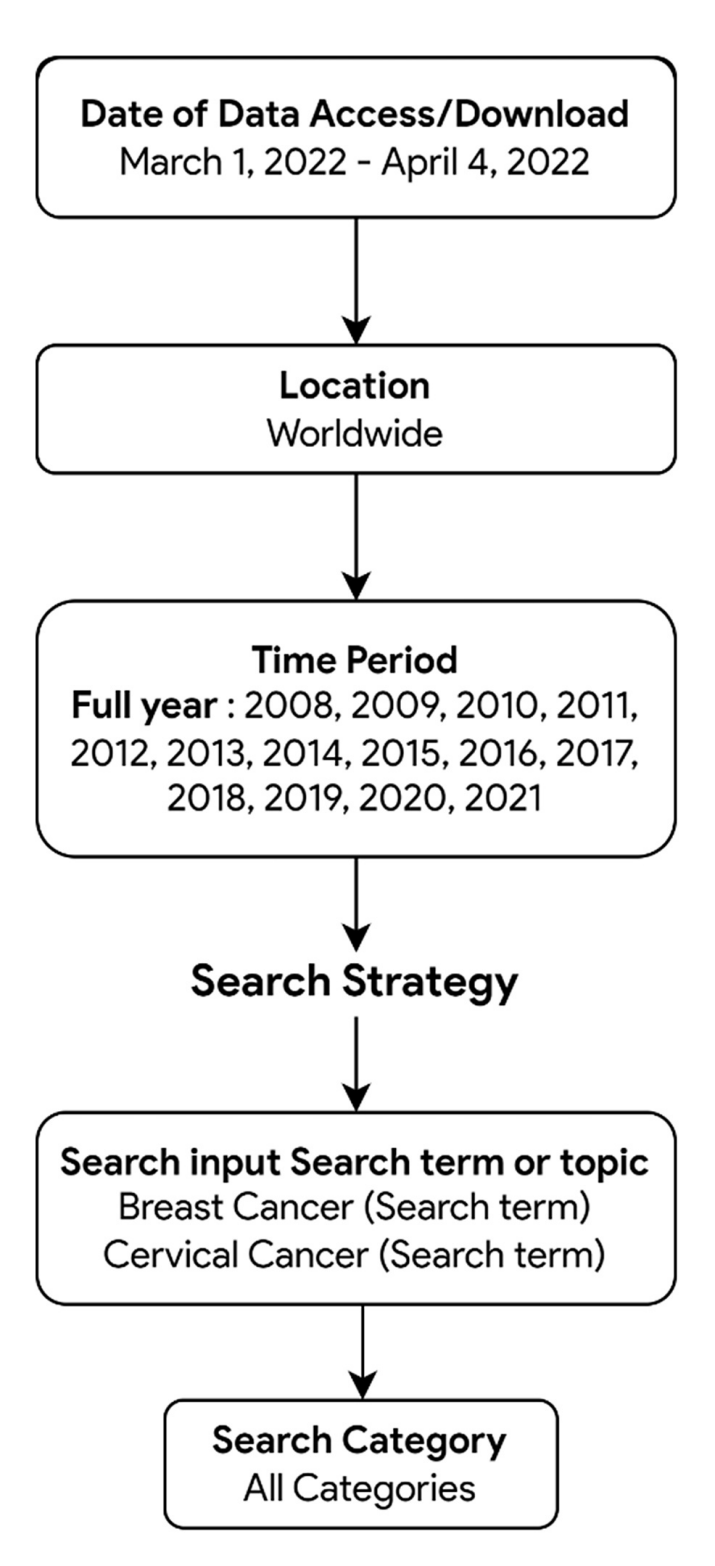
Google Trends search strategy

For this infodemiology study, we collected weekly and monthly search volumes of online information-seeking behaviors for the terms "breast cancer" and "cervical cancer" for each year from 2008 to 2021 and a 168-month period (January 2008 to December 2021) to assess the weekly and monthly trends of the RSVs. The data retrieval was conducted from March 1-April 4, 2022. To categorize the two cancer-related searches further, we performed additional searches using the search inputs that include "pink ribbon," "breast cancer screening," "mammogram", "cervical screening," "HPV infection," and "HPV vaccine". Figure 1 summarizes the search strategy using GT.

We collected weekly (52 or 53 weeks) RSVs of breast cancer and cervical cancer and analyzed public interest during BCAM (October), which begins in the 39th or 40th week of each year, and CCAM (January), which begins in the first week of each year. RSVs were pooled during the 168-month search.

Statistical analysis

We used a dot plot to present the "breast cancer" and "cervical cancer" monthly search trends from 2008 to 2021. We chose a joinpoint regression model using the Joinpoint Regression Program (version 4.9.1.0, April 2022, Statistical Research and Applications Branch, National Cancer Institute, MD, USA) to analyze time trends in GT RSVs data [20]. Joinpoint regression software analyzes trends by regression modeling while searching for temporal trend changes at time points called "joinpoints" and estimates regression models from previous joinpoints [21]. The number of joinpoints was obtained using a permutation model via the Monte Carlo resampling technique [21], and analysis criteria were set to find up to four joinpoints. The weekly or monthly (WPCs or MPCs) trend-change points were determined with 95% confidence intervals (CIs). The statistical significance was set at 0.05, indicating the slope was different from zero.

Ethical approval

We used the publicly available data published by GT (Google LLC, Mountain View, CA, USA). The study was approved by the institutional review board of Okayama University Hospital with a waiver for informed consent since the study intended to retrospectively analyze open data (No. 1910-009). All research methods were performed following relevant guidelines and regulations.

## Results

Breast cancer and cervical cancer online searches from 2008 to 2021 

Figure 1 depicts the annual changes in online searches for breast and cervical cancer from 2008 to 2021. Every year, breast cancer searches peak in October (BCAM). The highest RSV (100%) happened in October 2016 during BCAM, with a decreasing trend in 2020 (RSV: 61%) and 2021 (59%). During CCAM, which was in January, online searches for cervical cancer were over 90% in 2013, 2019, and 2020.

Weekly trends in online breast cancer search volumes from 2008 to 2021

Table 1 presents the trends and changes in the weekly RSVs for the search term "breast cancer" for each year from 2008 to 2021. There was a significant increase in RSV each year from the 36th to 37th week (mid-September) to the 39th to 40th week (the first week of BCAM, P<0.001) for breast cancer, ranging from 15.3% (95% CI: 8.6 to 22.4) in 2008 to 49.1% (95% CI: 24.6 to 78.4) in 2017. In 2020, however, the increase in RSV from the 37th week to the 40th week was not statistically significant at 22.4% (95% CI: -16.4 to 79.3). After the 40th week, the increase in RSV decreased by -22.5% (95% CI: -22.5 to -28.9) per week (P<0.001) until the 44th week. In the final quarter, from the 44th to the 52nd week, RSV decreased by -4.8% (95% CI: -6.5 to -3.0, P<0.001) per week, except for 2020, where the third quarter joinpoint was at the 40th week.

**Table 1:** Joinpoint regression analysis shows changes in breast cancer online search trends over time CI: confidence interval; WPC: weekly percentage change. *P<0.05 indicates that the WPC is significantly different from zero.

Breast cancer	Period 1		Period 2		Period 3		Period 4	
Year	Weeks	WPC (%) [95% CI]	Weeks	WPC (%) [95% CI]	Weeks	WPC (%) [95% CI]	Weeks	WPC (%) [95% CI]
2008	1 – 34	-0.4* [-0.8 – -0.1]	34 – 40	15.3* [8.6 – 22.4]	40 – 44	-14.5* [-25.2 – -2.3]	44 – 52	-6.4 [-9.1 – -3.6]
2009	1 – 36	-0.3* [-0.6 – -0.0]	36 – 40	27.4* [12.8 – 45.7]	40 – 45	-15.8* [-25.4 – -5.0]	45 – 52	-7.3* [-9.7 – -4.8]
2010	1 – 36	-0.8* [-1.1 – -0.4]	36 – 40	38.1* [18.2 – 61.3]	40 – 45	-18.8* [-26.4 – -10.4]	45 – 52	-5.5* [-9.4 – -1.5]
2011	1 – 34	-0.6 [-1.0 – -0.2]	34 – 41	21.4* [15.2 – 27.9]	41 – 45	-24.1* [-34.9 – -11.4]	45 – 52	-2.7 [-6.7 – 1.4]
2012	1 – 36	-0.5* [-0.9 – -0.2]	36 – 41	37.7* [24.6 – 52.3]	41 – 45	-30.0* [-40.2 – -17.9]	45 – 52	-5.2* [-9.1 – -1.1]
2013	1 – 37	0.0 [-0.2 – 0.3]	37 – 40	71.1* [34.7 – 117.2]	40 – 44	-31.3* [-39.1 – -22.6]	44 – 52	-4.2* [-6.7 – -1.7]
2014	1 – 36	0.0 [-0.3 – 0.3]	36 – 40	46.4* [30.6 – 64.2]	40 – 44	-30.4* [-37.9 – -22.0]	44 – 52	-4.6* [-7.0 – -2.2]
2015	1 – 36	0.2 [-0.1 – 0.5]	36 – 40	41.6* [25.7 – 59.5]	40 – 44	-26.5* [-34.8 – -17.2]	44 – 52	-5.6* [-8.0 – -3.1]
2016	1 – 36	0.0 [-0.2 – 0.3]	36 – 41	28.0* [21.2 – 35.2]	41 – 44	-32.4* [-43.2 – -19.6]	44 – 52	-3.3* [-5.1 – -1.5]
2017	1 – 37	-0.0 [-0.2 – 0.2]	37 – 40	49.1* [24.6 – 78.4]	40 – 45	-19.9* [-24.3 – -15.2]	45 – 52	-3.1* [-5.4 – -0.8]
2018	1 – 36	0.2 [-0.1 – 0.4]	36 – 40	32.5* [20.3 – 46.0]	40 – 44	-24.8* [-31.8 – -17.2]	44 – 52	-1.7 [-3.8 – 0.4]
2019	1 – 35	-0.1 [-0.4 – 0.1]	35 – 40	26.7* [19.0 – 34.8]	40 – 44	-23.5* [-30.7 – -15.6]	44 – 52	-2.1 [-4.2 – 0.0]
2020	1 – 15	-3.5* [-5.2 – -1.7]	15 – 37	1.5* [0.5 – 2.5]	37 – 40	22.4 [-16.4 – 79.3]	40 – 52	-8.1* [-10.2 – -6.0]
2021	1 – 37	0.2 [0.1 – 0.3]	37 – 40	39.9* [22.9 – 59.3]	40 – 45	-18.1* [-21.4 – -14.7]	45 – 52	-1.9* [-3.6 – -0.2]
Average	1 – 36	-0.2 [-0.4 – 0.0]	36 – 40	35.6* [24.5 – 47.8]	40 – 44	-22.5* [-22.5 – -28.9]	44 – 52	-4.8* [-6.5 – -3.0]

Weekly trends in online cervical cancer search volumes from 2008 to 2021

The trends and changes in the weekly RSVs for the search term "cervical cancer" from 2008 to 2021 are displayed in Table 2. There were multiple points with trend shifts, but no joinpoints were identified around the time of CCAM for cervical cancer. Briefly, significant trend changes were observed from week 1 to week 8 in 2019 (15.6%, 95% CI: 9.0 to 22.7), week 1 to week 23 in 2010 (2.6%, 95% CI: 1.3 to 3.9), and week 1 to week 11 in 2014 (2.5%, 95% CI: 1.1 to 4.0). In 2017, there was a 19.2% (95% CI: 9.1 to 30.1) per week increase in RSV during CCAM (weeks 1 to 4) for cervical cancer. Between late July (week 26) and mid-November (week 45), the RSV increased by 0.9% (95% CI: 0.6 to 1.3, P<0.001) per week.

**Table 2:** Joinpoint regression analysis shows changes in cervical cancer online search trends over time CI: confidence interval; WPC: weekly percentage change *P<0.05 indicates that the WPC is significantly different from zero.

Cervical cancer	Period 1		Period 2		Period 3		Period 4	
Year	Weeks	WPC (%) [95% CI]	Weeks	WPC (%) [95% CI]	Weeks	WPC (%) [95% CI]	Weeks	WPC (%) [95% CI]
2008	1 – 31	-0.3 [-0.7 – 0.1]	31 – 34	18.2* [-8.0 – 51.9]	34 – 52	-3.5* [-4.3 – -2.7]	-	-
2009	1 – 8	15.6* [9.0 – 22.7]	8 – 24	-5.2* [-7.0 – -3.4]	24 – 39	2.5* [0.4 – 4.7]	39 – 52	-3.2* [-5.5 – -1.0]
2010	1 – 12	2.6* [1.3 – 3.9]	12 – 21	-4.1 [-6.0 – -2.1]	21 – 45	1.0* [0.6 – 1.4]	45 – 52	-5.2* [-7.5 – -2.8]
2011	1 – 35	-0.2* [-0.4 – 0.0]	35 – 38	9.7 [-8.0 – 30.8]	38 – 49	-1.4* [-2.8 – 0.1]	49 – 52	-9.6* [-17.2 – -1.3]
2012	1 – 35	-0.7* [-1.0 – -0.3]	35 – 41	3.9 [-2.2 – 10.4]	41 – 52	-3.6* [-5.3 – -1.8]	-	-
2013	1 – 35	-0.3* [-0.5 – -0.1]	35 – 41	1.6 [-2.1 – 5.5]	41 – 48	-2.0 [-4.8 – 0.8]	48 – 52	-4.7 [-9.6 – 0.5]
2014	1 – 11	2.5* [1.1 – 4.0]	11 – 21	-1.6* [-3.2 – 0.0]	21 – 47	0.2 [-0.1 – 0.6]	47 – 52	-6.6* [-10.2 – -2.8]
2015	1 – 36	-0.2* [-0.4 – 0.0]	36 – 45	3.0* [0.9 – 5.1]	45 – 52	-5.1* [-7.4 – -2.7]	-	-
2016	1 – 49	-0.6* [-0.8 – -0.4]	49 – 52	-7.6 [-20.4 – 7.2]	-	-	-	-
2017	1 – 4	19.2* [9.1 – 30.1	4 – 7	-13.1 [-27.1 – 3.6]	7 – 49	-0.1 [-0.2 – 0.1]	49 – 52	-3.7 [-11.8 – 5.1]
2018	1 – 32	-0.6* [-0.9 – 0.4]	32 – 43	2.6* [1.1 – 4.1]	43 – 52	-4.2* [-5.8 – -2.5]	-	
2019	1 – 3	12.0 [-7.2 – 35.3]	3 – 21	-1.8* [-2.4 – -1.1]	21 – 45	0.6* [0.2 – 1.0]	45 – 52	-4.5* [-6.9 – -2.1]
2020	1 – 9	-1.1 [-3.3 – 1.2]	9 – 12	-17.3 [-32.9 – 1.9]	12 – 28	2.6* [1.7 – 3.5]	28 – 52	-0.3 [-0.7 – 0.1]
2021	1 – 34	-0.6* [-0.8 – -0.4]	34 – 44	2.2* [0.8 – 3.7]	44 – 52	-4.1* [-5.8 – -2.5]	-	-
Average	1 – 4	3.8 [-1.5 – 9.3]	4 – 26	-0.9* [-1.2 – -0.7]	26 – 45	0.9* [0.6 – 1.3]	45 – 52	-4.6* [-5.9 – -3.3]

Monthly trends in breast cancer and cervical cancer RSV between 2008 and 2021

**Figure 2:**
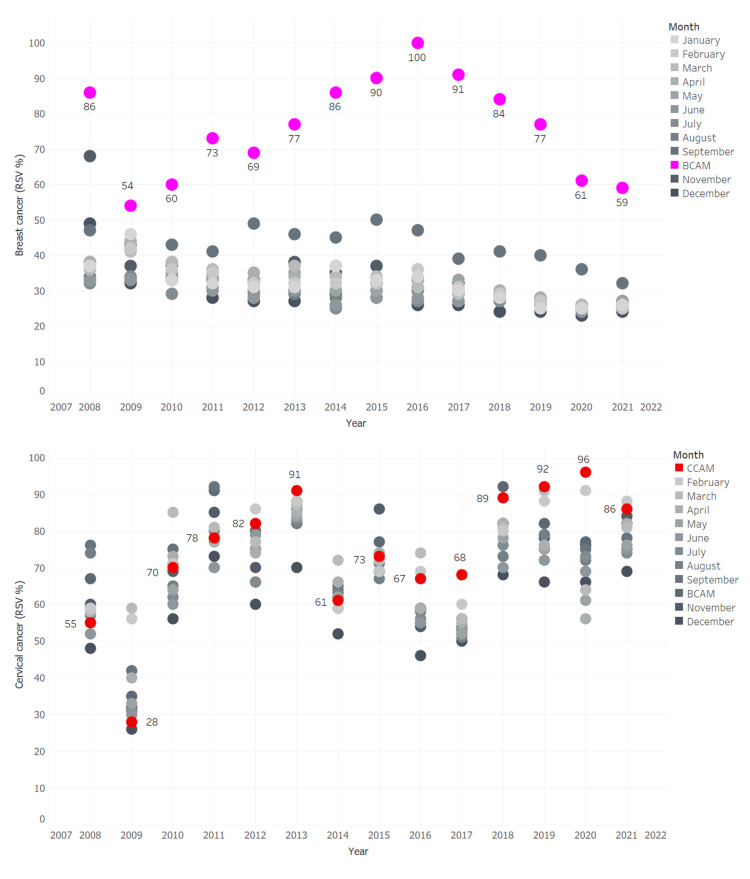
Worldwide trends in the public interest in “Breast cancer” and “Cervical cancer” from 2008 to 2021 BCAM: Breast cancer awareness month (October); CCAM: Cervical cancer awareness month (January)

**Figure 3:**
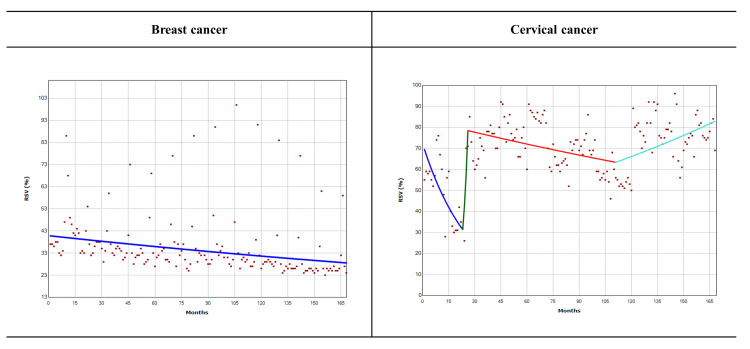
Global Trends in the monthly relative search volumes (%) of “Breast cancer” and “Cervical cancer” from 2008 to 2021 RSV: relative search volume Monthly RSV for the search terms” Breast cancer” and “Cervical cancer” between 2008 and 2021.

Figures 2, 3, and Table 3 show monthly RSV trends for breast and cervical cancer from 2008 to 2021. From 2008 to 2021, breast cancer RSV decreased every month, averaging -0.2% (95 percent CI: -0.3 to -0.1, P<0.001). RSV for cervical cancer increased by 0.5% (95% CI: 0.2 to 0.7, P<0.001) each month from May 2017 (month 112) to December 2021 (month 168).

**Table 3:** Joinpoint regression analysis showing monthly percentage changes in “breast cancer” and “cervical cancer” relative search volume on Google Trends from 2008 to 2021 Final selected model ^a^Breast cancer^ b^Cervical cancer *P<0.05 indicates that the monthly percentage change is significantly different from zero.

Model	Monthly percentage change (%)			
	Period (months)	Breast cancer	Period (months)	Cervical cancer
Model 1^a^	January 2008 – December 2021	-0.2* (-0.3 – -0.1)	January 2008 – December 2021	0.2* (0.1 – 0.3)
Model 2	January – October 2008	1.8 (-5.2 – 9.4)	January 2008 – October 2011	1.0* (0.5 – 1.5)
	October 2008 – December 2021	-0.2* (-0.3 – -0.1)	October 2011 – December 2021	0.0 (-0.1 – 0.1)
Model 3	January – November 2008	6.2 (-0.2 – 13.0)	January 2008 – November 2009	-3.5* (-4.6 – -2.4)
	November 2008 – March 2009	-9.0 (-39.1 – 36.2)	November 2009 – February 2010	31.1 (-19.5 – 113.5)
	March 2009 – December 2021	-0.2* (-0.3 – -0.1)	February 2010 – December 2021	0.0 (-0.1 – 0.1)
Model 4^b^	January 2008 – July 2008	-4.0 (-16.0 – 9.6)	January 2008 – November 2009	-3.5* (-4.6 – -2.5)
	July – October 2008	33.8 (-39.7 – 196.6)	November 2009 – February 2010	35.7 (-13.7 – 113.2)
	October 2008 – February 2009	-14.8 (-42.8 – 26.9)	February 2010 – May 2017	-0.3* (-0.4 – -0.1)
	February 2009 – December 2021	-0.2* (-0.3 – -0.1)	May 2017 – December 2021	0.5* (0.2 – 0.7)

## Discussion

Our study used GT data and joinpoint regression analysis to quantify the global public awareness of breast and cervical cancer using RSVs as surrogates. Our study demonstrated that BCAM had a significant yearly impact on global public interest in breast cancer from 2008 to 2021, while no significant increases in the trends were noted during CCAM. Interestingly, however, RSVs for breast cancer and related terms showed either significant declines or no changes through the period, while RSVs for cervical cancer significantly increased from 2017. RSVs generally declined for both breast and cervical cancer from 2019 to 2021, at the onset of the coronavirus 2019 (COVID-19) pandemic. These changes may reflect a global shift in focus away from cancer during the COVID-19 pandemic, especially given the significant global decrease in cancer screening services [22]. Combined, online interventions directly connecting people to preventive services during BCAM for breast cancer or throughout the year for cervical cancer using Google Advertisements (Ads) may be effective in improving access to primary prevention.

The World Health Organization (WHO) has made efforts to respond to the growing burden of breast cancer worldwide and the known outcome disparities, particularly by establishing the Global Breast Cancer Initiative (GBCI) [2]. The primary goal of GBCI is to reduce breast cancer mortality by 2.5% per year by three primary means: increase health promotion and early detection; reduce the diagnostic interval; and provide multidisciplinary, patient-centered care [2]. As part of GBCI, the International Agency for Research on Cancer (IARC) has taken the initiative to broaden the distribution of health-preventive breast cancer education globally, especially during BCAM, through social media, educational videos, and website infographics [23]. A prior systematic review demonstrated the efficacy of social media as a means of disseminating information, such as educational materials regarding cancer screening, though the evidence was notably limited [24]. Another study examined the impact of BCAM on public awareness of breast cancer in the United States, showing similar results to the present study with suggestions to employ online marketing and social media approaches during BCAM [25]. Online campaigns may also increase accessibility to preventive information related to breast cancer, especially in countries outside of the United States. For instance, preliminary findings from Mansour et al. suggest that social media can be a valuable tool for health promotion concerning screening programs across a variable group of people regardless of age, education level, or socioeconomic status [26].

Regarding cervical cancer, the WHO launched a global Cervical Cancer Elimination Initiative (CCEI), which utilizes a three-pronged “90-70-90” approach to fully vaccinate 90% of girls with the HPV vaccine by the age of 15 years, screen 70% of women by the age of 35 years and again by the age of 45 years, and treat 90% of women with precancerous lesions or invasive cancer [27,28]. As part of this initiative, IARC has also commented on the use of social media and online campaigns during CCAM [29]. However, given that there was no significant increase in RSVs during CCAM, the current approach to aggregating efforts during CCAM may not work out for cervical cancer, unlike breast cancer.

Our study quantified the extent of global public awareness of breast and cervical cancer in women worldwide by using GT data with joinpoint regression analysis to visualize trends in online searches pertinent to these diseases. Despite this strength, there are a few limitations that must be addressed. One limitation of this study is that the analysis of data trends is generalized rather than regionally characterized, which may mask disparities in trends in public awareness of breast cancer and cervical cancer across different areas of the world. Second, while BCAM and CCAM are currently recognized as worldwide health observances, they originally stemmed from the United States and might not be the most preferred search terms. Also, while the Google trend analysis has been utilized as a surrogate of public awareness and popularity, given the rise in internet use in scientific and medical communities, the results might not solely be representative of the interests of the general public and could be confounded. Also, given that we cannot discriminate where the Google searches came from, the present results need to be considered exploratory, and further research is warranted using quantifiable data tied to specific identification data such as searchers’ age, occupation, and context. Future studies may aim to better characterize differences in RSV trends between different countries or areas and direct initiatives to particular regions demonstrating poor public awareness of breast and cervical cancer. Also, future studies could examine changes in GT related to breast cancer and cervical cancer to determine the efficacy of increasing public awareness of these diseases with the more recent implementation of new global WHO public health initiatives such as GBCI for breast cancer and CCEI for cervical cancer. Should Google Ads be utilized by these global initiatives, further studies are warranted to assess their efficacy in promoting public awareness of breast and cervical cancer, including actual changes in the extent of health-improving behaviors such as engagement in mammography, a Pap test, and HPV vaccination rates. 

A previous pilot study utilizing Google Ads to discourage the use of tanning beds as a means of preventing skin cancer, though the data was inconclusive with regards to efficacy, demonstrated the feasibility of Google Ads to relay cancer preventive information to the public [30]. Given the results, a plausible online strategy may include a targeted approach to lead Google searches to actual preventive care. For breast cancer, health authorities or large regional healthcare networks can place Google Ads during BCAM that enable the public to access information about breast cancer prevention and to make an appointment for routine mammography. For cervical cancer, year-round Google Ads by regional health authorities or healthcare systems directly leading the public to make an appointment for HPV vaccines or a Pap smear may be considered a potential approach to impact the general public. Future pilot studies are warranted to see the effectiveness of the approach.

## Conclusions

Global trends in online searches related to breast cancer are observed to be high during BCAM from 2008 to 2021. Additionally, there generally appears to be an upward trend in searches related to cervical cancer from May 2017 to December 2021, with no particular significant increases during CCAM noted. The results can inform online initiatives and event-based opportunities such as BCAM and CCAM and the utilization of Google Ads to increase public awareness of breast and cervical cancer.
